# External validation of a Cox prognostic model: principles and methods

**DOI:** 10.1186/1471-2288-13-33

**Published:** 2013-03-06

**Authors:** Patrick Royston, Douglas G Altman

**Affiliations:** 1 Hub for Trials Methodology Research, MRC Clinical Trials Unit and University College London, Aviation House 125, Kingsway country London, WC2B 6NH UK; 2 Centre for Statistics in Medicine, University of Oxford Wolfson College, Linton Road, Oxford OX2 6UD, UK

**Keywords:** Time to event data, Prognostic models, Cox proportional hazards model, External validation, Discrimination, Calibration

## Abstract

**Background:**

A prognostic model should not enter clinical practice unless it has been demonstrated that it performs a useful role. External validation denotes evaluation of model performance in a sample independent of that used to develop the model. Unlike for logistic regression models, external validation of Cox models is sparsely treated in the literature. Successful validation of a model means achieving satisfactory discrimination and calibration (prediction accuracy) in the validation sample. Validating Cox models is not straightforward because event probabilities are estimated relative to an unspecified baseline function.

**Methods:**

We describe statistical approaches to external validation of a published Cox model according to the level of published information, specifically (1) the prognostic index only, (2) the prognostic index together with Kaplan-Meier curves for risk groups, and (3) the first two plus the baseline survival curve (the estimated survival function at the mean prognostic index across the sample). The most challenging task, requiring level 3 information, is assessing calibration, for which we suggest a method of approximating the baseline survival function.

**Results:**

We apply the methods to two comparable datasets in primary breast cancer, treating one as derivation and the other as validation sample. Results are presented for discrimination and calibration. We demonstrate plots of survival probabilities that can assist model evaluation.

**Conclusions:**

Our validation methods are applicable to a wide range of prognostic studies and provide researchers with a toolkit for external validation of a published Cox model.

## Background

Prognostic models, most of which are multivariable, have several critical applications. These include, for example, (i) informing treatment or other clinical decisions for individual patients, (ii) informing patients and their families about the likely course of a disease, (iii) creating clinical risk groups for informing treatment or for stratifying patients by disease severity in clinical trials, and (iv) risk adjustment when assessing the performance of health care systems [1]. See Moons et al [2] for a general discussion of prognostic research. It is widely accepted nowadays that a model should not enter clinical practice unless it has been demonstrated that it performs a useful role [3]. In reality, the criterion means that at the very least, a model should discriminate usefully between good and bad outcomes in patients whose data were not involved in the development of the model. In general, it is not essential to know how well a model performed in the derivation data, nor to match its performance in the derivation data. Evidence of performance comes from ‘validation studies’ [1]. For logistic regression models, for instance, there are several well-recognised approaches [4-9]. By contrast, the methodology for validation is not particularly well worked-out for models of time to event data. The main reason why time to event data provide a greater challenge is the almost invariable censoring of some observation times, caused by some patients’ outcomes remaining unascertained within the study period.

Before going further, we must carefully clarify the meaning of the term ‘validation’. Unfortunately, the term is vague in several respects. As Feinstein [10] pointed out, “Validation is one of those words … that is constantly used and seldom defined.” First, ‘validation’ may mean the *process of evaluating the performance *of a model, or it may mean *a successful outcome *of the aforementioned process [1]. Success would lead to a ‘validated’ model, i.e. one that is somehow certified as fit for purpose. Second, there are ‘flavours’ of validation, the primary distinction being between internal and external validation. Internal validation essentially means reusing parts or all of the dataset on which a model was developed to assess the likely overfit and correct for the resulting ‘optimism’ in the performance of the model. External validation means assessing the performance of a model already developed when applied to an independent dataset. By ‘independent’ we mean collected as part of an exercise separate from the development of the original model, for example by different investigators in a different geographical location. Some authors have distinguished between levels of stringency of validation, the most severe test being evaluation in dataset(s) that differ in investigators, location, and time period from those of the original model [1,11]. For example, models developed in academic centres may not apply in primary care settings due to differences in the case mix of patients; models built many years ago may not apply today because of improvements in treatment.

There are two fundamental aspects of evaluating and thus validating model performance: discrimination and calibration. Discrimination, sometimes known as ‘separation’, is the extent to which risk estimates from a model characterise different patient prognoses. Patients predicted to be at higher risk should exhibit higher event rates than those deemed at lower risk. Calibration reflects prediction accuracy. A well-calibrated risk score or prediction rule assigns the correct event probability at all levels of predicted risk. A miscalibrated rule under- or over-predicts the event probability, sometimes globally (‘miscalibration in the large’ [6]) and sometimes depending on the risk level or on specific covariates. Arguably, inadequate discrimination is a more important failing than poor calibration, since the latter can be improved by model recalibration [3,12], whereas the former cannot be altered. It is therefore particularly important to have measures that reliably identify poor discrimination. In essence, a validation study will assess the discrimination and calibration of a model on a new dataset.

Nowadays, researchers predominantly use the Cox proportional hazards model to analyse time to event data [13,14]. We are concerned here with external validation of a published Cox proportional hazards model developed in a single dataset and evaluated in one or more independent datasets. We take the perspective of a researcher who does not have access to the original data but who has an independent sample on which to evaluate the model’s performance.

A Cox model estimates hazard ratios, which measure how much a covariate affects the hazard function for the event of interest. According to the proportional hazards assumption, covariates act multiplicatively on a baseline hazard function. The latter is usually defined as the hazard function for which all covariate values are zero. Throughout the present paper, we take it as the hazard function at the mean value of all the covariates, or equivalently, at the mean value of the prognostic index. Given the baseline hazard function, the baseline survival curve and survival curve for any given covariate pattern can be estimated.

A Cox model supports estimation of relative differences in risk between patients with different characteristics, but since it does not estimate the baseline hazard function *per se*, it does not estimate absolute risks (event probabilities) or absolute differences in prognosis. By contrast, parametric models, although much less popular, are fully specified and therefore *can* estimate absolute risks and survival probabilities [12,15]. Published results from Cox models are usually restricted to the regression coefficients (log hazard ratios) and their standard errors or confidence intervals. Nevertheless, there is still much that can be done in validating Cox models. The issue of external validation, which seems to have been largely neglected in the literature, forms the main focus of the present paper.

The structure of the paper is as follows. First we describe two breast cancer datasets used to exemplify a multivariable prognostic model developed on one clinical dataset and evaluated on another. This section also gives details of the candidate prognostic variables and the resulting model. In the section ‘Products of a Cox model’, we describe key quantities relating to validation that may be derived by fitting a Cox model. We next identify three increasingly detailed levels of information from the derivation dataset that support different aspects of validation. In the section ‘Validation’ we discuss techniques of validation, and we then apply the techniques to the breast cancer datasets. The Discussion section includes comments on the interpretation of validation findings.

## Methods

### Breast cancer datasets and model

#### Derivation and validation data

The original dataset comprised 2982 primary breast cancer patients whose records were included in the Rotterdam tumour bank, of whom 1546 had node-positive disease. For details, see Reference [16]. Follow-up time ranged from 1 to 231 months (median 107 months). The outcome, recurrence-free survival time (RFS), was defined as the time from primary surgery to the earlier of disease recurrence or death from any cause. To reduce the possible influence of long-term survivors on parameter estimates in a Cox model [17], we censored events occurring after 84 months, the maximum follow-up time in the validation dataset. In a real study this would presumably not be done, but it has little influence on the results. Since the validation dataset comprised only node-positive patients and nodal status is an important prognostic factor, we omitted the node-negative patients to create the derivation dataset. With the RFS outcome, 965 (i.e. nearly 90 percent) of events were observed in the period from 0 to 84 months out of a possible 1080 available with the full follow-up time of 231 months. The Rotterdam data (with RFS truncated to 120 months) can be downloaded from the following URL: http://www.stata-press.com/data/fpsaus.html. The Stata data file is called rott2.dta.

The validation data were taken from a trial in primary breast cancer. From July 1984 to December 1989, the German Breast Cancer Study Group (GBSG) recruited 720 patients with primary node positive breast cancer into a factorial 2 × 2 design to investigate the effectiveness of three versus six cycles of chemotherapy and of additional hormonal treatment with tamoxifen [18]. The dataset we use comprises the recurrence-free survival (RFS) time of the 686 patients (with 299 events) who had complete data on several standard prognostic variables. The definition of RFS was the same as for the derivation dataset. The maximum follow-up time available was 7 years. The dataset can be loaded into Stata via the command webuse brcancer. Note that an internet connection is needed for this to work.

#### Prognostic factors

Candidate prognostic variables in the breast cancer datasets were age at primary surgery (age, years), menopausal status (meno, 0 = premenopausal, 1 = postmenopausal), tumour size (size), tumour grade (grade), number of positive lymph nodes (nodes), progesterone receptors (pgr, fmol/l), oestrogen receptors (er, fmol/l), hormonal treatment (hormon, 0 = no, 1 = yes), and chemotherapy (chemo). Tumour size (mm) was not available as a continuous variable in the Rotterdam dataset, therefore a standard coding was used; the base category was ≤ 20 mm and two dummy variables were used, namely 20 to 50 mm (sized1) and > 50 mm (sized2). We excluded grade, since it was measured according to a different protocol in the two datasets, and chemo, since all patients in the validation dataset received chemotherapy.

The distribution of the remaining candidate predictors is shown in Table 1. The distributions happen to be broadly similar between the derivation and validation datasets, although this is not a requirement for successful validation. Differences in the receptor variables, pgr and er, may be due to variation in the methods of measurement.

**Table 1:** Candidate prognostic factors in the derivation and validation datasets

**Variable name**	**Variable**	**(Definition/units)**		***n *(%) or mean (SD)**		
			**Derivation**		**Validation**	
Tumour size	sized0	(≤20 mm)	501	(32*%*)	180	(26*%*)
	sized1	(20 to 50 mm)	783	(51*%*)	453	(66*%*)
	sized2	(>50 mm)	262	(17*%*)	53	(8*%*)
Menopausal status	meno	(premenopausal)	628	(41*%*)	290	(42*%*)
		(postmenopausal)	918	(59*%*)	396	(58*%*)
Hormone treatment	hormon	(no)	1207	(78*%*)	440	(64*%*)
		(yes)	339	(22%)	246	(36%)
Age	age	(years)	56.0	(13.0)	53.1	(10.1)
Positive lymph nodes	nodes	(number)	5.2	(4.9)	5.0	(5.5)
Progesterone recep.	pgr	(fmol/l)	156	(299)	110	(202)
Oestrogen recep.	er	(fmol/l)	165	(267)	96	(153)

Values in parentheses are category percentages for categorical variables, and SD for continuous variables. Recep. = receptors.

#### Model

We developed a multivariable Cox model for the Rotterdam (derivation) data by applying the multivariable fractional polynomial (MFP) model-building procedure [19] to the candidate variables (see Table 1). We selected variables by backward elimination and fractional polynomial (FP) functions using a 5% significance level. The resulting model comprised age (FP2 function, powers 3,3), meno, sized1, sized2, nodes^-0.5^, er (linear function) and hormon. See Table 2 for details. The covariate pgr was not significant at the 5% level and was eliminated. The FP2(3,3) transformation of age yields the covariates age^3^ and age^3^× ln(age).

**Table 2:** Multivariable prognostic model for the derivation dataset

**Variable**	$\hat{\beta }$	**SE**
age ^3^	1.07	0.40
age ^3 ^× ln (age)	9.13	2.64
meno	0.46	0.12
sized1	0.23	0.08
sized2	0.31	0.08
nodes^-0.5^	-1.74	0.14
er	-0.34	0.13
hormon	-0.35	0.08

Note that for legibility of the regression coefficients, age was divided by 100 and er by 1000 before analysis.

### Products of a Cox model

The baseline hazard function is a vital component of the Cox model. However, with the partial likelihood method, it is not estimated. For practical purposes, it is reasonable to consider the Cox model as consisting of its regression coefficients and their covariance matrix. Often, the $\hat{\beta }$ values and their standard errors are regarded as the Cox model. Application of the model requires ancillary quantities, in particular, the prognostic index and the baseline survival function. We address these aspects in turn.

#### Prognostic index

For practical application, the main product of a Cox model is a prognostic index (PI). The variables which comprise the PI may have been selected by a multivariable modelling technique that automatically takes into account correlations among variables, such as stepwise selection from a list of candidate variables. This approach often removes apparently redundant predictors. Some of the variables included in a PI may be continuous, and some may be transformed (as with the MFP method, or when using regression splines [20]). There is no necessity to dichotomise continuous variables—indeed, it wastes information [21].

The obvious way to construct a PI is to take the linear predictor from a Cox model. The linear predictor is a weighted sum of the variables in the model, where the weights are the regression coefficients. In the usual situation in which the event of interest is an adverse outcome, high values indicate a worse prognosis. The PI for an individual is then the log relative hazard compared with a hypothetical individual whose PI is zero.

Approaches other than simply using the linear predictor from a Cox model are surprisingly common. In a recent survey, Mallett et al [22] reviewed a sample of 43 studies which used regression methods. Most researchers (38/43) developed a multivariable prognostic model (almost always a Cox model), but 9/38 did not report the coefficients to enable a PI to be calculated. In 11 studies, however, after obtaining their model the authors modified either the choice of variables or their weighting. In particular, in four studies the authors simply counted the number of risk factors present out of those determined as important in the model, i.e. all variables were taken to be equally important. Indeed, sometimes such scores are derived without fitting a model at all [23,24].

Regardless of the justification for any *post hoc *manipulation, the approach to validation of a model is the same, no matter how a PI was obtained. It is, however, worth mentioning some factors that increase the likelihood of poor performance in a validation study: stepwise selection of variables from a large number of candidate variables with a small number of events, data-dependent cutpoint selection for continuous predictors, extensive missing data, a differently defined endpoint. In cancer, for example, the endpoint could be overall survival in the derivation dataset and progression-free survival in the validation dataset.

#### Example

**Figure 1:**
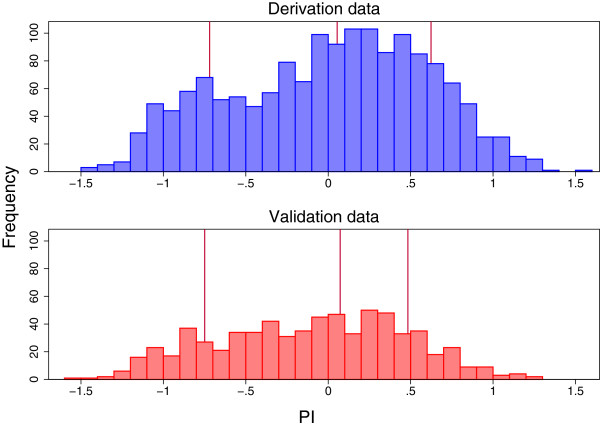
**Histogram of the PI in the derivation and validation datasets. **The PI was centered on the mean in the derivation dataset. The vertical lines show the 16th, 50th and 84th centiles of the PI in each dataset.

Figure 1 shows the distribution of the PI in the two datasets. We recommend producing such a plot for the validation dataset (acknowledging, of course, that in our paradigm the derivation data are supposed to be unavailable). The histogram shows the general level of the log relative hazard and indicates the spread. The spread (standard deviation), * s*say, is functionally related to the explained variation statistic $R_{\text{PM}}^{2}$[25] via $R_{\text{PM}}^{2}=s^{2}/\left(\sigma^{2}+s^{2}\right)$, where *σ*^2^ plays the role of the residual variance in linear regression. For proportional hazards models, *σ*^2 ^= *Π*^2^/6 ≃ 1.645. A related measure is $R_{D}^{2}$[26], based on *D*, a measure of the ability of a model to discriminate between good and poor patient outcomes (see section ‘Measures of discrimination’). The plot may also exhibit outliers, which can be caused by inappropriate extrapolation of the relative hazard, for example when predicting with a function of continuous covariates such as an FP or spline. In the present example there are no obvious outliers or other irregularities.

#### Survival probabilities

As already stated, a Cox model comprises an unestimated baseline hazard function, *h*_0_(*t*) say, and a linear predictor or PI whose regression coefficients are estimated from the data. The hazard function is modelled as *h*(*t*) = *h*_0_(*t*) exp(PI). The Cox model is sometimes called ‘semi-parametric’ because the linear predictor is fully parametric, whereas the baseline hazard function is quite general (‘non-parametric’): it can be any positive-valued function of time. If we knew the baseline hazard function, we could integrate it over time to compute the cumulative hazard function, $H_{0}(t)=\overset{t}{\underset{0}{\int }}h_{0}(u)\mathrm{du}$, and hence the baseline survival function, *S*_0_(*t*) = exp [-*H*_0_(*t*)]. A well-known non-parametric estimate of the baseline survival function is available [27], but it is impracticable to report the result concisely. Consequently, as lamented by van Houwelingen [12], the baseline survival function is almost never given by authors of published medical articles that report a Cox model.

#### Risk groups

Mainly for clinical and statistical reasons, a PI is often used to create risk groups. The statistical reason is that it facilitates the comparison of actual survival probabilites with model-based estimates. Kaplan-Meier curves for each risk group offer an intuitive depiction of variation in prognosis. The disadvantage of grouping is that associated with any categorisation of a continuous variable [21]: information is lost, particularly at extreme risks. An alternative approach is to derive survival curves directly from the Cox model; a comparison with the Kaplan-Meier curves gives one possible assessment of the model calibration.

#### Example

We took the PI for the derivation data as the linear predictor from the multivariable Cox model reported in Table 2. We centered the PI on ‘average risk’ by subtracting the mean of -1.32 [28]. The same value was subtracted from the PI evaluated for all individuals in the validation dataset. To create prognostic groups, we categorised the PI into 4 groups at the 16th, 50th and 84th centiles in the Rotterdam data (see the section ‘L2: PI and Kaplan-Meier curves for risk groups’ for the rationale for this choice of cut-points), giving 2 smaller groups at relatively low and high risk of recurrence or death, respectively, and two larger, central groups at lower or higher intermediate risk. We call these groups the Good, Fairly good, Fairly poor and Poor risk groups. The percentages of patients in these four groups in the validation dataset are 18.1*%*, 40.4*%*, 32.8*%* and 8.8*%*, respectively, showing overall a slightly better prognosis than in the derivation dataset. The mean (SD) of the PI is 0.00(0.60) in the derivation dataset and -0.11(0.57) in the validation dataset.

### Levels of information from the derivation dataset

We assume that a model has been developed on a derivation dataset and published in the medical literature. We have to hand a potential validation dataset in which we wish to evaluate the performance of the published model. We do not assume access to the individual patient data from the derivation study. We cannot therefore re-fit or adjust the original model in any detailed way. A key element in the ensuing discussion is the prognostic index (PI), the weighted combination of risk factors comprising the model. Given the PI, the weights of the individual predictors are unimportant. Options for validation depend on the information available in the publication describing the multivariable model for the derivation dataset. We consider the following 3 levels of information: 

• Cox model yielding PI, i.e. a set of regression coefficients

• L1 plus risk groups plus Kaplan-Meier curves

• L2 plus baseline survival curve

We consider in a structured way which aspects of the model can be validated for each level of information available for the derivation dataset. We illustrate the approaches in the breast cancer datasets.

#### L1: Prognostic index only

The minimum information needed to apply a model and for its validation is reported coefficients (i.e. weights) for each variable included in the model. The precise coding of each covariate is required. A table of hazard ratios is acceptable, since the required weights can be derived by taking logarithms. Authors may have expressed the combination of variables as an equation (PI), but this is not essential.

#### L2: PI and Kaplan-Meier curves for risk groups

When a PI has been developed, a common practice is to form risk groups by categorising the PI, and then to superimpose Kaplan-Meier survival curves for the groups in a single graph. Mallett et al [22] (Table 2) found three or four groups to be the most common. However, there is no consensus in the literature on (a) how many risk groups should be created, and (b) where (and why) to position the cutpoints [29]. Developing sensible guidance for choosing risk groups remains a topic for further research.

Nevertheless, statistical common sense dictates that a modest number of risk groups (say, 5 or fewer) is preferable to a large number. Two groups is likely to be too few to satisfy the needs of clinical practice and research applications. With a large number, the survival curves may be unstable and the discrimination between neighbouring groups is likely to be poor. Unequal group sizes seem preferable to equal groups, since they enable identification of patients with more extreme prognoses and group together patients with largely similar prognoses.

For present purposes, to give a reasonable spread of risk we chose to work with four prognostic groups, using cut-points on the PI determined by Cox’s method [30]. For a given number of groups, the method is designed to minimise the loss of information that occurs with grouping. The required cut-points are the 16th, 50th and 84th centiles of the continuous variable, here the PI in the derivation dataset. On a standard Normal scale, these correspond to 0 and approximately ±1, i.e. mean ± 1SD. See further discussion of the grouping issue in the section ‘Grouped versus individual prognostication’.

#### L3: L2 information and baseline survival function

The baseline survival function is crucial, since it encapsulates the information needed to assess calibration of survival probabilities in the derivation dataset, and more importantly, calibration in validation datasets. Later, we describe a simple method of determining a smooth, parametric baseline survival function in the derivation dataset which can be reported in publications and transported to other datasets. When an estimate of the baseline survival function has been obtained, calibration can be assessed. Without the baseline survival function, it is not possible to judge how good the calibration in an independent sample is.

### Validation

#### Data: Basics and reporting requirements

We assume that the derivation and validation datasets are basically compatible. This requires some preliminary evaluation of the available variables. A further important issue to consider when conducting a validation study is the comparability of the patients and settings with those included in the derivation study. We do not address the issue in this paper. There is also a need for an adequate description of the sample, e.g. dates of collection of information, inclusion or exclusion criteria, treatment information, location, investigators, and so on. Another consideration is the length of follow-up in the validation sample relative to the derivation sample.

In all cases, the guiding principle is to apply the model in exactly the same way in the validation data as in the derivation data, or at least to be aware of any differences. In practice, we require reporting of the definition of each covariate (including its units) and the outcome variable in the derivation dataset, and precisely how the variables were treated in the development and final expression of the model. Categorical variables may be coded in different ways. For example, sparsely populated categories may be combined, and ordinal variables may be coded so as to respect the ordering (see Reference [19], 55–56). Continuous variables may be categorised or transformed, as with methods based on FP functions or regression splines.

#### Validation options

Table 3 lays out seven suggestions for external validation of models for time to event data. These are discussed individually below. The column headed ‘Aspect’ indicates whether a given method addresses the discrimination (D), calibration (C) or more general fit (F) of a model.

**Table 3:** Some methods for external validation and the level of information about the derivation study they require

	**Method**	**Aspect**	**Information**		
			**L1**	**L2**	**L3**
1	Regression on PI in validation data	D, F	✓	✓	✓
2	Check model misspecification/fit	F	✓	✓	✓
3	Measures of discrimination	D	✓	✓	✓
4	Kaplan-Meier curves for risk groups	D, C	×	✓	✓
5	Logrank/Cox tests between risk groups	D	×	✓	✓
6	Hazard ratios across risk groups	D	×	✓	✓
7	Calibration	C	×	×	✓

L1, L2 and L3 denote increasing amounts of information (see text). Key to Aspect: D, discrimination; C, calibration; F, model fit.

As can be seen from Table 3, most of the methods address discrimination. Calibration is harder to assess and requires a higher level of information about the derivation study.

#### Regression on the PI

A recognised approach to validation is to estimate the regression coefficient on the PI or risk score in the validation dataset [4,12], sometimes known as the ‘calibration slope’. The PI or risk score must be computed in the validation dataset exactly as reported for the derivation dataset. There must be no refitting of regression coefficients, for example. When the PI is the linear predictor from a model (the usual situation), the calibration slope is by construction exactly 1 in the derivation dataset. Subject to one caveat (see below), the discrimination in the validation dataset is about the same when the slope on the PI is approximately 1. If the slope in the validation dataset is < 1, discrimination is poorer, and conversely if it is > 1, discrimination is better. A likelihood-ratio test that the slope is 1 is straightforward to perform. Note that the resulting *P*-value may be anti-conservative, since it does not allow for uncertainty in the estimated regression coefficients that constitute the PI.

The caveat concerns case mix. If the prognosis of the patients in the validation sample is less heterogeneous than in the derivation sample, the spread (SD) of the PI will be less. This will be reflected in smaller explained variation and other discrimination measures [31].

#### Check model misspecification/fit

One reason why the slope on the PI may differ from 1 in the validation dataset is that the regression coefficients for one or more covariates may differ between the datasets. This can be tested formally (ignoring uncertainty of estimates in the derivation dataset) by running a Cox regression on the covariates **x **in the validation dataset, ‘offsetting’ the original PI evaluated in the validation dataset. The corresponding model is

$$\ln h\left(t\right)=\ln h_{0}\left(t\right)+x^{\prime }\beta^{*}+\text{PI}$$

The coefficient of PI is constrained to equal 1. By construction, the *β*^∗ ^values are differences between the *β*s estimated in the model fitted to the derivation dataset (treated as fixed numbers) and those estimated in the model fitted to the validation dataset (treated as random quantities). It may in principle be possible to allow for uncertainty in the estimate of *β *in the derivation dataset when testing *β*^∗^. However, this would require the variance-covariate matrix of $\hat{\beta }$, something which is rarely if ever reported and something which would extend the level of information needed about the derivation model.

From the point of view of successful validation, the ‘best’ result is that all the coefficients *β*^∗ ^are 0. A joint test of *β*^∗ ^= **0 **may be applied. A separate test could be applied to each coefficient; however, the joint test provides some protection against inflation of the type 1 error.

While such an approach can be informative, it immediately raises the question of what to do if lack of fit is found for one or more variables (i.e. if ${\hat{\beta }}^{*}$ is incompatible with 0). If individual patient data are available for the derivation dataset (not considered here), it may be possible to go back and improve the original model, but that is not the aim of a validation exercise. Sometimes, lack of fit may be caused by differences in the definition, measurement or even units of variables between the two datasets.

Similarly, one can investigate other aspects of fit of the PI in the validation sample, for example, the validity of the proportional hazards assumption. However, the same caveat applies. Even if the proportional hazards assumption is untenable, a model may still provide good discrimination (see section ‘Measures of discrimination’), but the calibration would need to be scrutinised. Such a ‘partially validated’ model may perform well enough to retain clinical utility.

See the section ‘Method 2: Check model misspecification/fit’ for an example.

#### Measures of discrimination

In simple terms, discrimination for time to event models reflects separation between survival curves for individuals or groups. Maintaining discrimination in independent data is a key principle of model validation. Discrimination can be assessed in different ways. Over the last 20 years, several measures have been proposed. While a comprehensive review of methods is beyond the present scope, some recent descriptions and comparisons of measures may be found in [32,33].

The main requirement for a measure of discrimination to be useful in external validation is that it be evaluable in the validation dataset. A measure whose definition does not directly involve the response variable (outcome) is not eligible. An example of this is the explained-variation statistic $R_{\text{PM}}^{2}$[25], which is a monotone transformation of the variance of the PI across patients, as mentioned above. The outcome is needed in the calculation of the PI and hence $R_{\text{PM}}^{2}$ in the derivation dataset, but the PI and $R_{\text{PM}}^{2}$ can be calculated independently of the outcome in the validation dataset.

Here, for the purpose of illustration we focus on three measures: Harrell’s *c*-index of concordance [34], Gönen and Heller’s unbiased concordance statistic *K*[35], and the Royston-Sauerbrei *D* statistic [26]. We emphasised that many other measures are available, including for example the Brier score [36], and more recently, Zheng et al [37]’s positive predictive value approach, but they do not change the general principles.

Harrell’s *c*-index is defined as the proportion of all patient pairs in which the predictions and outcomes are concordant. (Because of censoring, not all pairs are evaluable [34].) Gönen and Heller’s *K *statistic is an extension to time-to-event data of the well-known area under the ROC curve which is used to assess the discrimination of logistic regression models. It involves only the regression parameters and the covariate distribution and is therefore asymptotically unbiased.

Royston and Sauerbrei [26] proposed $R_{D}^{2}$ as a measure of explained variation on the log relative hazard scale based on the authors’ *D *statistic. *D *measures prognostic separation of survival curves, and is closely related to the standard deviation of the prognostic index. It is computed by ordering the PI across patients, calculating the rankits (expected standard normal order statistics) corresponding to these values, dividing the latter by a factor $\kappa =\sqrt{8/\Pi }≃1.596$ and performing Cox regression on the scaled rankits. The resulting regression coefficient is *D*. The conversion to $R_{D}^{2}$ is given by where (as in the section ‘Prognostic index’) *σ*^2 ^= *Π*^2^/6 ≃ 1.645.

$$R_{D}^{2}=\frac{D^{2}/\kappa^{2}}{\sigma^{2}+D^{2}/\kappa^{2}}$$

Since the *c*, *D *and $R_{D}^{2}$ measures depend only on the rank of the estimated relative hazard, all are easily determined from the PI evaluated in the validation dataset. No new model needs to be fitted in the validation dataset, either to the PI or to the original variables. Of course, a comparison with the derivation dataset can only be made if the same measures were reported for that dataset. They cannot be obtained retrospectively unless individual patient data are available for the derivation dataset. Even if they were not originally reported, however, there is value in reporting them as part of the validation exercise. They require only L1 information.

#### Kaplan-Meier curves for risk groups

As discussed in the section ‘Risk groups’, having developed a prognostic model, many authors present survival curves for risk groups derived after categorising the PI. Here we discuss the use of such survival curves in assessing model discrimination and calibration. L2 information is required.

##### Discrimination

Kaplan-Meier survival curves for risk groups provide informal evidence of discrimination. The more widely separated are the curves, the better is the discrimination. A Kaplan-Meier graph for both datasets allows a visual comparison of discrimination between datasets. We strongly recommend producing such plots.

As already noted, an issue is that the case mixes of the derivation and validation datasets may differ markedly. The term case mix refers to the type or mix of patients treated by a hospital or unit. Even if the model is ‘correct’, the Kaplan-Meier curves for a given risk group may suffer from a type of residual confounding and may also differ across datasets. Residual confounding (a term from epidemiology) occurs when the relationship between the outcome and the PI is not fully accounted for by categorisation of the data into prognostic groups; some inhomogeneity of prognosis remains within groups. Therefore, a naïve comparison between Kaplan-Meier curves for the two datasets could be misleading. Residual confounding is reduced if a larger number of risk groups is created, but having too many groups brings its own dangers.

##### Calibration

Calibration describes how accurately the estimates or predictions of survival from a model reflect the survival in the observed data [3,9]. Miscalibration is a type of bias. For a given PI value, a comprehensive and accurate PI should yield a similar level of risk over time and thus similar survival curves in the derivation and validation datasets—that is, it should be well calibrated. A comparison of Kaplan-Meier plots supports a rough assessment of model calibration. Good calibration may be inferred if the survival curves for a given risk group agree well between the derivation and validation datasets.

The calibration assessment provided by comparing Kaplan-Meier curves between datasets is not a strict comparison between observed and predicted values, however, since the Cox model is not being used directly to predict survival probabilities. Instead, the PI from the Cox model provides only a rank ordering of risk, from which risk groups are created and corresponding survival probabilities are estimated by the Kaplan-Meier method. A direct type of calibration assessment requires L3 information and is discussed in the section ‘Calibration’.

#### Logrank or Cox tests between risk groups

As just noted, larger separation between survival curves represents better discrimination. Some analysts perform logrank or Cox tests between risk groups, inferring successful validation if statistical significance is ‘achieved’ in the validation dataset. *We do not recommend this approach*, and we mention it only because it is sometimes done. Such *P*-values do not quantify discrimination; they quantify the evidence against a silly null hypothesis, namely that survival of the risk groups coincides. It is analogous to assessing the significance of the correlation between methods of measurement in a method-comparison study [38], where the inappropriate null hypothesis is of no association between two methods that are supposed to measure the same thing.

#### Hazard ratios across risk groups

In contrast to *P*-values for comparing risk groups, evaluating hazard ratios is a sensible check of discrimination. If two survival curves are more widely separated, the hazard ratio tends to be larger. A table of (log) hazard ratios and their SEs or CIs may be a useful accompaniment to the Kaplan-Meier curves discussed above. Such a table may be obtained after fitting a Cox model with a dummy variable representing each risk group (except for a reference group). See Table 4 for an example. In reality, such hazard ratios are not often reported.

**Table 4:** Discrimination measures and hazard ratios evaluated in the derivation and validation datasets

**Measure**	**Derivation data**		**Validation data**	
	**Estimate**	**SE**	**Estimate**	**SE**
Harrell *c*-index	0.673	0.009	0.658	0.016
Gönen & Heller *K*	0.655	0.008	0.645	0.015
Explained variation ($R_{D}^{2}$)	0.166	0.017	0.145	0.033
HR: group 2 versus 1	1.58	0.19	1.63	0.35
HR: group 3 versus 1	3.33	0.39	3.34	0.70
HR: group 4 versus 1	4.75	0.60	6.33	1.54

All values are based on the PI of the prognostic model. Risk groups were defined as described in the text. Standard errors (SE) for the *c*-index and for *RD2 *were estimated from 200 bootstrap samples. HR = hazard ratio.

#### Calibration

Earlier, we discussed an assessment of calibration through risk groups (requiring L2 information). Here, we propose a stricter form of calibration requiring data from individuals (L3 information). To our knowledge, L3 information is never published, so this stricter type of calibration assessment is never done.

When an estimate of the baseline survival function in the derivation dataset is available, it is possible to check the strict calibration of a Cox model [12]. (Even values at a limited number of time-points may be utilised.) Given an estimate of the baseline survival function, *S*_0_(*t*), we combine *S*_0_(*t*) with the PI to predict survival probabilities. To avoid the need for extrapolation of the baseline survival curve beyond the observed range of *t*, we should ensure that the follow-up period in the validation dataset is no longer than that in the derivation dataset. If necessary, the follow-up time in the validation dataset should be truncated at or before the maximum in the derivation dataset.

Since we are assuming L3 information, we take it that *S*_0_(*t*) has been estimated in the derivation dataset and is explicitly available, e.g. as an approximating mathematical function or a look-up table (even for a few time points). See the section ‘Method 7: Calibration and the baseline hazard function’ for a suggestion to use fractional polynomials to approximate *S*_0_(*t*). A procedure for checking calibration graphically is as follows: 

1. If the model is ‘correct’, the baseline survival function (i.e. covariate-adjusted survival) should be similar across datasets. We calculate *S*_0_(*t*) in the validation dataset, either directly from our mathematical approximation or for example, by linear interpolation of *S*_0_(*t*) at the observed times in the validation dataset.

2. For a given value PI _*i*_, we compute the predicted survival function in the validation dataset as $S^{\text{val}}\left(t;{\text{PI}}_{i}\right)=S_{0}{\left(t\right)}^{\text{exp}\left({\text{PI}}_{i}\right)}$.

3. We average the curves *S*^val^(*t*;PI_*i*_) over all members of each risk group at the observed times in the validation dataset to obtain the expected survival curve in each group.

4. In a graph, we superimpose the expected and observed (Kaplan-Meier) survival curves for each risk group. (We can, of course, also present the expected and observed survival probabilities in tabular form at specific time-points).

Note that although steps 1 and 2 are performed at the individual level, steps 3 and 4 require risk groups. Assessing calibration of survival probabilities at the individual level appears impracticable.

Miscalibration may appear in different guises in the plot. For example, miscalibration in the large would present as a general under- or over-estimation of survival probabilities. Alternatively, we may see inaccurate predictions at particular times or in particular risk groups.

Finally, it may be helpful to investigate the accuracy of the baseline survival function itself. We assume that a simple mathematical approximation to the baseline survival function in the derivation dataset is available. This curve should apply to the validation dataset too. How well it fits can be evaluated by comparing it with a Kaplan-Meier-like estimate of the baseline survival function in the validation data. The latter may be obtained by standard methods after fitting a Cox model to the validation data with no covariates other than the PI with regression coefficient constrained to 1 (i.e. the model ln*h*(*t*) = ln*h*_0_(*t*)+ PI). This approach preserves the PI from the derivation dataset without re-estimating any regression coefficients. A large difference between the two estimates of the baseline survival function suggests some fundamental miscalibration of the model and therefore a failure of validation.

## Results

### Illustrative example

We present an example of the seven options identified for validation (Table 3) by re-analysis of the Rotterdam and GBSG datasets.

#### Method 1: Regression on PI in validation data

The slope in a Cox model on the PI in the validation dataset is 0.97 (SE 0.11). The slope is close to 1 and is not significantly different from 1 (*P *= 0.8), so the discrimination seems to be preserved.

#### Method 2: Check model misspecification/fit

There is no overall evidence of lack of fit of the PI in the validation dataset, as a joint test of all the predictors is non-significant ($\chi_{8}^{2}=6.08,P=0.6$) in the model with the PI offset (see the section ‘Check model misspecification/fit’ for details). A standard test of the PH assumption for the model in the validation dataset with a single predictor (the PI) using scaled Schoenfeld residuals suggests some breach of the PH assumption (*P *= 0.03). The interpretation is that the regression coefficient for the PI changes somewhat over time in the validation dataset. This is not a major concern, however, since the overall calibration slope is near 1 (see above) and the discrimination of the model is largely preserved (see below).

#### Method 3: Measures of discrimination

Harrell’s *c*-index, Gönen and Heller’s *K *statistic and Royston & Sauerbrei’s $R_{D}^{2}$ are given for both datasets in the first three rows of Table 4. The discrimination is modest, but not unusually so for this particular setting. The values of *c*,*K* and of $R_{D}^{2}$ are similar in size in each dataset (but exhibiting a slight reduction in the validation sample), showing good validation (performance) of the model. Note that *K* is somewhat smaller than *c *in each case, to be expected since *c* is known to be be biased away from the null due to right-censoring of times to event.

#### Method 4: Kaplan-Meier curves for risk groups

**Figure 2:**
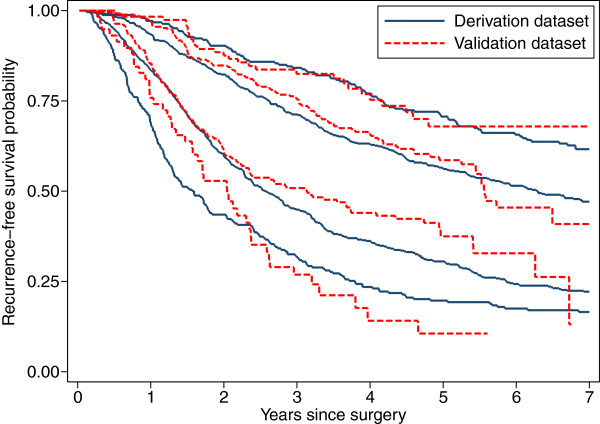
Kaplan-Meier curves for recurrence-free survival in 4 risk groups in the derivation and validation datasets, based on an MFP model.

Figure 2 shows Kaplan-Meier curves for recurrence-free survival in the two datasets according to risk group. We have shown both sets of curves on the same plot, but in practice, visual comparison of the validation results with the original publication may be needed. Inspection of the plot yields the following information: 

1. Both sets of four curves are quite well separated, confirming our earlier conclusion that the model has some discrimination in both datasets.

2. The discrimination in the derivation and validation datasets is broadly similar, but the model seems less able to distinguish between the two intermediate risk groups in the validation dataset. 

3. The survival curves in the validation dataset do not agree perfectly with those in the derivation dataset in the early follow-up phase, up to about 2 years. Subjectively, therefore, there appears to be a degree of miscalibration.

#### Method 5: Logrank or Cox P-values

As already discussed, we deprecate this method and therefore do not present detailed results for it. In any case, it is clear from Table 4 that the log hazard ratios comparing risk groups are significantly different from zero in the validation dataset.

#### Method 6: Hazard ratios between risk groups

Hazard ratios between risk groups are presented in Table 4. The hazard ratios seen in the derivation dataset are well-maintained in the validation dataset, confirming the impression in Figure 2.

#### Method 7: Calibration and the baseline hazard function

**Figure 3:**
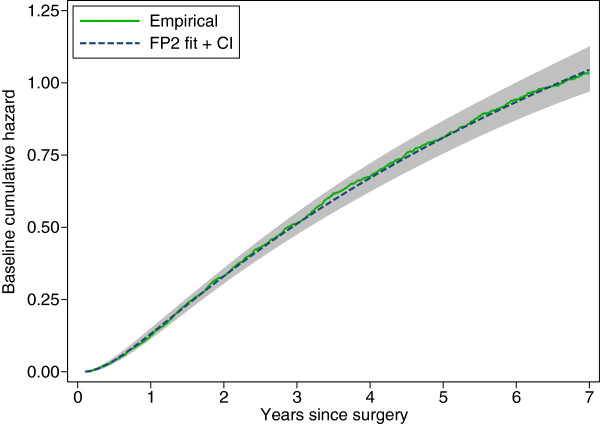
**Baseline cumulative hazard function in the derivation dataset. **Jagged curve, empirical (Kaplan-Meier-like) estimate; smooth line and grey band, FP2 fit with pointwise 95% confidence interval determined by bootstrap resampling.

We applied the graphical and analytic methods described in the section ‘Calibration’ to the PI. To illustrate one possible approach, we used fractional polynomial regression to approximate the log baseline cumulative hazard function, ln*H*_0_(*t*), as a smooth function of time in the derivation dataset. With ordinary least squares estimation, we obtained the following approximations:

$$\begin{array}{ll} \ln H_{0}\left(t\right)=1.727-3.759t^{-0.5}-0.356t^{-0.5}\ln t\; & \;\\ S_{0}\left(t\right)\;=\text{exp}\left[-\text{exp}\left(\ln H_{0}\left(t\right)\right)\right]\; & \; \end{array}$$

i.e., an FP2 function with powers (-0.5,-0.5). The curve is an excellent fit to ln*H*_0_(*t*) in the derivation dataset, explaining 99.9*% *of the variation (see Figure 3). The pointwise 95% confidence interval was obtained by taking 100 bootstrap samples of the derivation data, fitting a Cox model to the PI in each sample, predicting the log cumulative hazard function, finding the best-fitting FP2 function by regression on time, and computing the pointwise standard deviation across the bootstrap samples. The CI was calculated on the log scale and back-transformed to the cumulative hazard scale to give the results shown in Figure 3.

**Figure 4:**
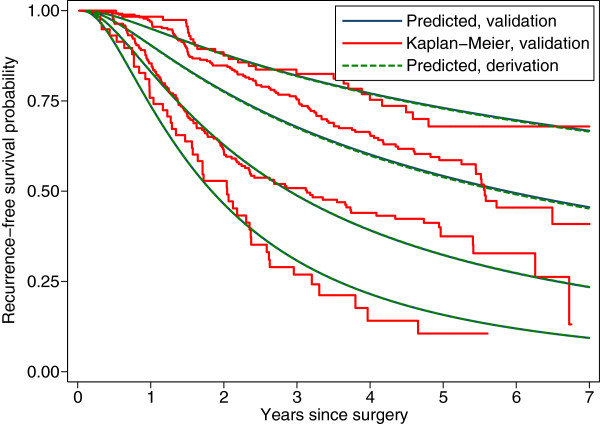
**Calibration of survival probabilities in the validation dataset. **Smooth lines: recurrence-free survival as predicted in the derivation and validation datasets from the PI and the smoothed baseline survival function from the derivation dataset. Jagged lines: Kaplan-Meier estimates in the three risk groups in the validation dataset. Note that the pairs of smooth curves for the two highest risk groups happen nearly to coincide and are visually indistinguishable.

**Figure 5:**
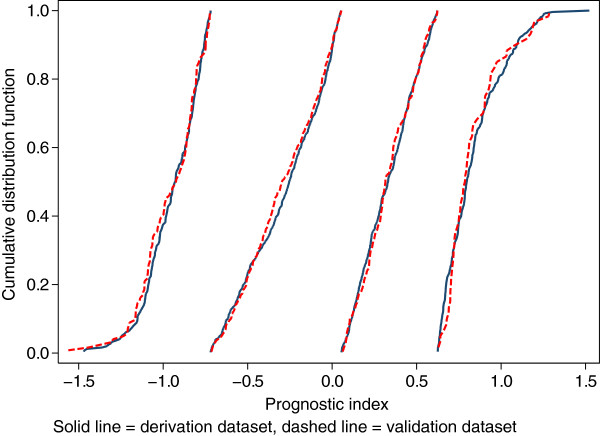
**Empirical cumulative distribution functions of the PI by dataset and risk group.** Solid lines, derivation data; dashed lines, validation data.

We applied the averaging method to obtain predicted mean survival curves in the validation dataset. The predicted mean survival curves are compared with the Kaplan-Meier survival curves in the three risk groups in Figure 4. We see that the calibration is reasonable for all except the Fairly good risk group, where the model consistently underpredicts. The predicted survival curves in the two datasets (smooth lines) are almost identical, reflecting the similarity in the distributions of the PI in each risk group across the datasets. Figure 5 shows the cumulative distribution function (c.d.f.) of the PI by dataset and risk group; the c.d.f.’s within risk groups are nearly superimposable.

Two- and five-year recurrence-free survival probabilities according to risk group in the validation dataset are shown in Table 5. The tabulated values are a subset of those presented graphically in Figures 2 and 4. Differences between observed ($\hat{S}\left(t\right)$) and predicted ($\bar{S}\left(t\right)$) survival probabilities are apparent, but are not substantial.

**Table 5:** Calibration by risk group

**Risk group**	***t***	**Kaplan-Meier**								**Predicted**
	**(yr)**	**Derivation**				**Validation**				**Validation**
		***N***	**Ev.**	$\hat{S}(t)$	**SE**	***N***	**Ev.**	$\hat{S}(t)$	**SE**	$\bar{S}(t)$
1. Good	2	247	90	0.90	0.02	124	28	0.88	0.03	0.88
	5			0.71	0.03			0.68	0.05	0.73
2. Fairly good	2	526	270	0.82	0.02	277	103	0.85	0.02	0.78
	5			0.56	0.02			0.59	0.03	0.54
3. Fairly poor	2	526	401	0.60	0.02	225	123	0.61	0.03	0.63
	5			0.31	0.02			0.37	0.04	0.32
4. Poor	2	247	204	0.44	0.03	60	45	0.53	0.07	0.47
	5			0.20	0.03			0.11	0.05	0.16

Values shown are Kaplan-Meier estimates and standard errors of recurrence-free survival probabilties in the three groups at two times. Values labelled Predicted were predicted from the derivation dataset by applying the PI to the smooth baseline survival estimate at the individual level in the validation dataset, and averaging across each risk group. *N *and Ev. denote the number of patients and events in each group.

**Figure 6:**
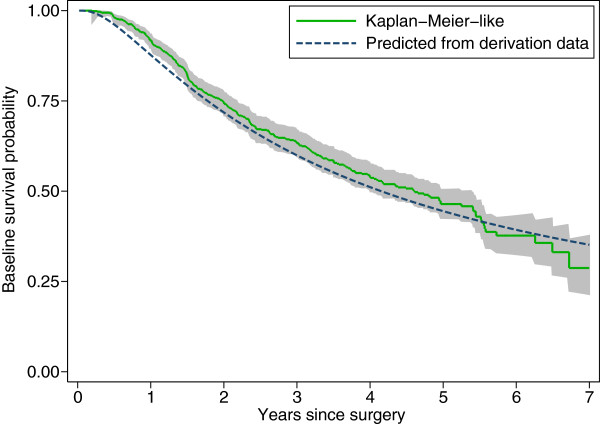
**Estimates of the baseline survival function in the validation dataset.** The grey region is a pointwise 95% confidence interval based on bootstrap resampling. Set text for details.

Finally, Figure 6 compares two different estimates of the baseline survival function in the validation dataset. The empirical estimate is accompanied by a pointwise 95% confidence interval based on bootstrap resampling of the FP2 fit. The smooth, solid line is the FP2 approximation to *S*_0_(*t*) obtained from the derivation dataset, as described above. The irregular line is the Kaplan-Meier-like estimate of the baseline survival function estimated after fitting the model *h *(*t*; PI) = *h*_0 _(*t*) exp(PI) to the validation data. The two estimates are similar, but not identical. Particularly in the early follow-up phase, prognosis seems to be slightly better in the validation dataset, after adjusting for the PI.

## Discussion

### Why is validation important?

A prognostic model that appears to predict outcome well on a derivation dataset but which predicts weakly or not at all on a plausibly related independent dataset is clearly not ‘fit for purpose’, whatever purpose may be envisaged. External validation is an essential first step towards acceptance of a model into clinical practice. Taking a wider view, model validation is a type of replication of a research result. The need for replication of an important finding is a cornerstone of the scientific method and is accepted practice in all areas of science, including clinical trials. Sometimes multiple replications may be demanded, which in our context means more than one attempt at model validation. Certain well-used prognostic models, for example in breast cancer and in heart disease, have indeed been validated several times in different populations.

### Grouped versus individual prognostication

Our method 4 and the requirement for L2 information involves defining prognostic groups, typically (though not necessarily) by polytomising the prognostic index. However, the application of a continuous score to estimate the prognostic index and hence the outlook of a given patient does not require grouping and may be preferred by some as the primary way to apply the model. We are not arguing for grouping as advantageous in the interpretation or *application* of the model, nor of course in the reporting of the model where we point to the need for presenting both the prognostic index and the baseline survival function. We more use grouping as a technical device to indicate how well the model fits and predicts in a validation sample, for example by graphing or tabulating performance. Also, there is a distinction perhaps between the individual perspective (clinical application) and the broader population perspective (assessment of outcomes in population groups), a contrast that arises in many areas.

Technically, it may be preferable to estimate calibration curves for survival probabilities without the use of risk groups. An approach in this direction is provided by the routines calibrate and val.surv in the *R* package rms[39]. The rms package implements many of the procedures described in Harrell’s book [6].

### Interpreting results of the validation exercise

To discuss findings, and therefore to evaluate how successfully a model has been ‘validated’, we need a context in which the prognostic model is to be applied. In other words, we need to know the purpose(s) for which the model was developed. As examples, we consider two general scenarios: risk stratification, e.g. as a criterion for determining entry to clinical trials, or in adjusting outcomes for disease severity when evaluating performance of health care institutions; and individual risk prediction, for physicians to advise patients and possibly to determine treatment options, or for direct use by patients for their own information.

For risk stratification purposes, discrimination is clearly the key indicator of model success or failure, although calibration still plays a role. In the breast cancer example, Table 4 and Figure 2 show that the model performs about as well in the validation dataset as in the derivation dataset, and has acceptable calibration (since adequate risk stratification is more important than absolute prediction accuracy). One cannot really ask for more. Nevertheless, in absolute terms, the discrimination is modest; a *c*-index of <0.7 and an explained variation of under 20% are not large. This is not an issue specific to the validation process, but is a consideration for deciding whether a (validated) model is useful or not. However, even a model that stratifies risk relatively weakly may be better than no model.

As Henderson & Keiding [40] point out, prognostic models are not good at individual predictions of outcome. Nevertheless, we can interpret graphs like Figures 2 and 4 in the following manner. Prognosis is clearly worst in the Poor group and best in the Good group, and this is confirmed in the validation dataset. Event times for individuals vary greatly, even for a given PI value. One can state, however, that there is about a 50:50 chance of an event within the first 2 years for a patient in the Poor group and 5 years in the Fairly good group (median time to event). We cannot estimate the median time to event in the Good group because the follow-up period is too short. A minor failing of the model in the validation sample is in predicting short term outcomes (1-2 years). The model tends to underestimate the time to event, that is actual prognosis is better than the model suggests. Further investigation beyond the scope of the paper suggests that failure of the proportional hazards assumption might be one reason why the short-term predictions are rather ‘off’.

Our proposed assessments show that the discrimination of the Rotterdam model is similar across the two datasets, and that the calibration is reasonably good. This implies that the evaluation of the model comes out reasonably satisfactorily. Crudely speaking, “the Rotterdam model validates quite well”.

### What is not validation?

In our view, the following practices that we have seen in the literature do not constitute validation of a model: 

• *Repeating the whole modelling process on new data*. This could involve comparing variables selected in the model, comparing regression coefficients and assessing goodness of fit. The result would be a new model, not validation of an existing one.

• *Refitting to the validation data the variables in the final model from the derivation data*. Again, this could involve comparing regression coefficients and gauging goodness of fit, and again the result would be a new model, albeit with the same predictors.

• *Calculating the PI from the original model and fitting this single predictor to the validation data*. This is one of the options in Table 3 but does not in itself constitute validation. If the calibration slope was much different from 1, there might be a case for proposing the recalibrated PI as an updated or revised model.

To repeat, to us the process of validation means assessing the performance of a predefined model in new data. It does not mean tinkering with the original model, which comes under the heading of ‘updating’ or ‘revising’ a model. All three approaches lead to a new model rather than a validation of the existing model, and as such, would themselves need external validation.

### What can be done if validation fails?

If a model ‘fails to validate’, for example it has adequate discrimination but poor calibration, we may wish to refine it. What can be done? Whereas methodology exists and has been applied to logistic regression models [41,42], the methods do not transfer to Cox models. An approach to recalibration and minimal updating of a model based on the Weibull distribution is described by van Houwelingen [12] (details not given). Of course, an updated model needs to be validated.

### Validating risk groups

We have so far considered the external validation of a Cox model that yields a continuous PI. However, key parts of our assessments have relied on the availability of risk groups. An extension of our approach could be to regard the risk groups themselves as of primary interest, rather than as an adjustable by-product of a PI. Risk groups can be derived, as we have done, from a continuous PI, or by another method. Possibilities include neural nets, various classification methods (e.g. support vector machines), CART, counting risk factors, or indeed any method that can define mutually exclusive groups.

Having defined risk groups, a Cox model can be estimated based on a dummy variable for each group, as in the section ‘Hazard ratios across risk groups’. The methods we describe in Table 3 can be applied to validate this simplified model. This implies that unambiguous rules exist for defining the risk groups in an independent sample.

The main advantage of this approach is applicability to a wide variety of risk ‘engines’. When a continuous PI is available (but is ‘discarded’ in favour of groups), however, disadvantages are loss of information and loss of flexibility in applications. A given prognostic classification scheme may be too crude for some types of application. For example, it may be important to identify patients with a very good or very poor prognosis, but working only with a specific scheme may preclude it.

### Sample size considerations

As can be seen in Table 4, a discrimination measure such as $R_{D}^{2}$ may have fairly large uncertainty, even with a moderate number of patients and events (686 and 299, respectively, in the validation dataset). It is clear, therefore, that a substantial validation sample is required to provide a reasonably precise estimate of such a measure. Validating a model on a few tens of patients can never suffice. Work is ongoing to develop reliable methods for calculating the necessary sample size for validation studies in terms of the magnitude of the *D* or $R_{D}^{2}$ measure and its desired precision [43]. Dunkler *et al*[44] used bootstrap resampling to investigate the impact of increasing the sample size on the precision of a measure of explained variation.

### Implications for reporting

We hope that we have made a convincing case for reporting at least up to L3 information for the derivation dataset and its Cox model. L1 and L2 information are quite often reported, but L3 is extremely rare. Nevertheless, without L3 information we can’t properly assess the calibration of a model. One option might be to contact the original investigators and request a suitable estimate of the baseline survival function. As we have shown, obtaining a simple but adequate approximation to the baseline survival function is not difficult, and indeed can be tackled in other ways if desired (e.g. spline functions [45]). Ideally, the entire derivation dataset (i.e. ‘L4’ information) and all its details would be made publicly available to facilitate validation of a proposed model. Such a paradigm does not seem to be likely to become common, except possibly in genomic studies where original study data are quite often placed in a data repository.

### Applicability to extended Cox models

Our sequence of validation steps requires a prognostic index that can be used to assign a relative hazard to every patient that does not change over time. In essence, this is the proportional hazards assumption. Extended Cox models, for example with time-dependent effects in which some of the regression coefficients change over time, do not satisfy the PH assumption and cannot be validated according to our scheme. Stratified Cox models also present a challenge to strict assessment of calibration in a validation dataset, since there is no longer a unique baseline survival function. However, assessing the discrimination of a stratified model is straightforward.

## Conclusions

In contrast to the logistic regression case, little attention has been accorded in the literature to validation of Cox models, despite their ubiquity. We believe that more attention should be given to validation of Cox models, especially in the common case where only L2 information about the derivation data has been published. The most fruitful approach relies on the comparison of Kaplan-Meier curves.

We intend to write a report for the Stata Journal describing the calculations needed to implement our approach to external validation.

In summary, we have discussed the importance of validation and have provided guidance for those who wish to evaluate the performance of a Cox model in a new dataset. However, judging adequacy of model performance in new data remains challenging.

## Abbreviations

CART: classification and regression tree; CI: confidence interval; FP: fractional polynomial; GBSG: German Breast Cancer Study Group; L1, L2, L3: Level 1, 2, 3 (information); MFP: multivariable fractional polynomial; PH: proportional hazards; PI: prognostic index; RFS: recurrence-free survival.

## Competing interests

The authors declare that they have no competing interests.

## Authors’ contributions

PR and DGA jointly originated the methodology and both contributed to drafting of the article. PR performed the statistical analysis and prepared the manuscript, including figures and tables. Both authors read and approved the final manuscript.

## Authors’ information

Both authors are biostatisticians.

## Pre-publication history

The pre-publication history for this paper can be accessed here:

http://www.biomedcentral.com/1471-2288/13/33/prepub
